# Antiretroviral therapy adherence and its determinant factors among people living with HIV/AIDS: a case study in Iran

**DOI:** 10.1186/s13104-019-4204-5

**Published:** 2019-03-22

**Authors:** Mohammad Ali Morowatisharifabad, Ehsan Movahed, Jamileh Farokhzadian, Ruhollah Nikooie, Mahdieh Hosseinzadeh, Mohsen Askarishahi, Reza Bidaki

**Affiliations:** 1 Elderly Health Research Center, School of Public Health, Shahid Sadoughi University of Medical Sciences, Yazd, Iran; 2 Health Education and Health Promotion, School of Public Health, Shahid Sadoughi University of Medical Sciences, Yazd, Iran; 3 Nursing Research Center, Kerman University of Medical Sciences, Kerman, Iran; 4 Department of Exercise Physiology, Faculty of Sports Sciences, Shahid Bahonar University, Kerman, Iran; 5 Nutrition and Food Security Research Center, Shahid Sadoughi University of Medical Sciences, Yazd, Iran; 6 Department of Biostatistics and Epidemiology, Shahid Sadoughi University of Medical Sciences, Yazd, Iran; 7 Research Center of Addiction and Behavioral Sciences & Diabetes Research Center, Shahid Sadoughi University of Medical Sciences, Yazd, Iran

**Keywords:** ART adherence, HIV/AIDS, Determinant factors

## Abstract

**Objectives:**

This descriptive-correlational study was conducted on 122 Iranian people living with HIV (PWHIV), who referred to a behavioral diseases counseling center in 2018. The AIDS Clinical Trial Group (ACTG) questionnaire was used to collect the required data. The study aimed to determine the level of medication adherence and its determinants in PWHIV.

**Results:**

About 75.4% (confidence interval 67.2%–82.8%) of the samples had a good combined antiretroviral therapy (cART) adherence and 74.6% (n = 91) of them were sure about the positive effects of medications on their health. Patients reported that most important reasons for medication non-adherence included forgetfulness, high drug dosage, lack of knowledge about ART value, and transportation problems.

## Introduction

The worldwide implementation of antiretroviral therapy (ART) has increased the survival rate of people living with HIV (PWHIV), so that the disease condition has shifted from a fatal to a chronic disease [1]. According to the latest reported statistics, 21.7 million people received ART drugs up to 2017 [2]. The number of HIV patients in Iran was reported as 66,000 in 2016. Moreover, the number of new cases of HIV infections has increased 21% since 2010, but the AIDS mortality rate decreased 14% [2]. Failure to ART adherence can cause dangerous outcomes such as substance abuse, depression, and spread of infections such as hepatitis. The ART adherence can reduce the symptoms of HIV, enhance the immunological body function, and increase the include physician–patient interaction, drug-related side effects, healthcare systems, and some patient-related factors such as age, regimens, academic education level, race/ethnicity, and smoking longevity [3].

As indicated in the literature, the ART adherence rate of more than 95% is required to prevent the virus proliferation and ensure its complete containment [4, 5]. A study on PWHIV in India in 2016 showed that the ART adherence was at a low level [5]. Moreover, the ART adherence among PWHIV in Tehran, Brazil, and Nepal was 59.5 to 94.8% [4, 6, 7].

Effective factors on the ART adherence [8]. The results of a study by Bam et al. indicated that the transportation problems and lack of knowledge led to the treatment non-adherence in PWHIV [6]. Experience of HIV-related rejections, non-acceptance, and discrimination reduced the patients’ motivation to maintain their optimal levels of health [9]. People who live in smaller communities experience more problems in this regard than the residents of larger cities. Therefore, it is difficult for PWHIV to use the health care services freely. These patients are usually concerned about the disclosure of their disease and its consequences; therefore, they prefer to use self-medication and do not receive health care services [10].

Given the prevalence of HIV/AIDS and the importance of ART for PWHIV, ART adherence seems to be the best and most effective way to improve these patients’ life quality. In addition, a limited number of studies were conducted over this subject in Iran. Therefore, the present research was carried out to investigate combined antiretroviral therapy (cART) adherence and its effective factors in HIV positive patients.

## Main text

### Materials and methods

#### The study and setting

This cross-sectional study was conducted on patients with HIV/AIDS, who were over 18 years of age. These patients referred to the behavioral diseases counseling center of Iran in 2018.

#### Sampling

The inclusion criteria for the study participants were having 18 years or more and using ART for 6 months. Since the statistical population included 183 individuals, census sampling method was conducted. Considering that 122 people participated in the study, the response rate was calculated as 66.67%.

#### Data collection

The AIDS Clinical Trial Group (ACTG) questionnaire, designed by Kekwaletswe for HIV positive patients, was applied in this study. The questionnaire consists of six parts. The first part relates to the ART adherence and includes two questions: (1) loss of ART within a week and (2) loss of ART within the past month (How many pills have you lost in a month or a week?). The adherence rate was calculated by multiplying the number of pills consumed per week or month by 100 divided by the total received tablets. The cutoff point of adherence was considered at 95%. In this study, the mean adherence of seven and 30 days were considered as the ART adherence. The second part of the questionnaire deals with the causes of ART non-adherence and includes 18 items (never = 0, rarely = 1, sometimes = 2, and often = 3). The third part of ACTG investigates 20 common symptoms experienced by patients during the past month (never = 0, rarely = 1, sometimes = 2, and often = 3). The fourth part of the questionnaire studies the self-efficacy of adherence and beliefs with regard to the usefulness of drugs. This section contains three items, which should be answered on a 4-point Likert scale (ranging from “I’m not sure at all = 0” to “I am completely sure = 3”). The fifth part considers social support. In this section, participants’ satisfaction with family and friends’ support was studied in one item (very dissatisfied = 0 to very satisfied = 3). The patients’ satisfaction with the family and friends’ help in taking ART was also evaluated in one item (not at all = 0 to very much = 4). Part six was about alcohol consumption (in the last 30 days) and drug abuse (in the last 6 months), which should be answered on a seven-point Lickert scale (never = zero, once a month = 1, two or three times a month = 2, once or twice a week = 3, three or four times a week = 4, almost every day = 5, and daily = 6). Kekwaletswe et al. confirmed the validity and reliability of this questionnaire by Cronbach’s alpha (0.71) [11, 12]. To conduct this study in the Iranian culture and among the people of Kerman, the necessary adjustments were made in the ACTG questionnaire. Its content validity was confirmed by a panel of expert containing 10 physicians and infection specialists in Kerman University of Medical Sciences. The reliability of the adjusted questionnaire was also calculated as 0.79 by Cronbach alpha.

#### Statistical analysis

Descriptive statistics were applied to describe the characteristics of the study population, their ART adherence, reasons for non-adherence, symptoms, alcohol consumption, and drug abuse. Univariate and multivariate logistic regressions were run to investigate the predictive factors of the ART adherence. To analyze the data, SPSS version 24 was used.

### Results

A total of 122 PWHIV with an average age of 41.88 ± 9.46 years participated in the study; 53.3% of the participants were male, 46.7% were married, and 36.9% had a diploma or higher educational degrees. The findings showed that 54.1% of patients were unemployed and 41.8% had an income of over US $ 60. The CD4+ T cell measure was more than 350 in 59% of the participants. The viral load was less than 50 in 63.9% of cases and 41.8% of the patients did not mention any risk factors such as the drug abuse. Furthermore, 57.4% of individuals had no history of methadone use and 34.4% of them had no symptoms during the last month.

The mean ART adherence was 91.86 ± 20.81; 24.6% had weak adherence and 75.4% had good adherence. Most patients (74.6%) believed that ART had a positive effect on their health and 59% of them were also confident that lack of ART would increase the resistance of HIV against drugs. In addition, 67.2% of participants were satisfied with the support of other people. In this regard, 71.3% of patients mentioned that their family members and friends supported them to take ART.

Forgetfulness in 26.7% of the cases was the cause of ART non-adherence. Moreover, a high number of ART, lack of knowledge about the medication’s worth, and transportation problems (each with 13.3%) were the main reasons for ART non-adherence (Table 1).

**Table 1:** Frequency distribution of the reason for no medication taking in the research units with poor medication adherence (n = 30)

Phrase	Sometimes/often	
	n	%
I was away from home	2	6.7
I was very busy (I had more important things to do)	2	6.7
I forgot	8	26.7
There were many pills	4	13.3
I did not understand the value of the drugs	4	13.3
I wanted to get away from the side effects of medications	1	3.3
I did not want others to realize that I’m taking medicine	0	0
I wanted to make a difference in my daily life	3	10.0
I felt that drugs were harmful and toxic	3	10.0
I felt very drowsy	2	6.7
I was sick (my condition got worse)	3	10.0
I had transportation problems	4	13.3
I felt depressed	4	13.3
Taking pills in certain meals (with a meal, or with an empty stomach, etc.) confused me.	1	3.3
I lost my medications	2	6.7
The pills were destroyed due to hot weather	0	0
I was tired of taking pills (I felt disable)	1	3.3
I had a good feeling and thought that I was healthy	3	10.0

The results of univariate logistic regression showed a significant relationship between the emergence of symptoms in the last 4 weeks and the ART adherence. Furthermore, the odds of poor adherence was 3.63 times higher in participants who had symptoms in the last 4 weeks than the asymptomatic ones (P = 0.02, CI = 1.24–10.62) (Table 2). The ART adherence did not have any relationship with other variables.

**Table 2:** The univariate and multivariate logistic regression models of the medication adherence with the study variables

Characteristic	Univariate logistic regression			Multivariate logistic regression		
	Odds ratio	Confidence interval	P value	Odds ratio	Confidence interval	P value
Age	0.99	0.95–1.04	0.75			
*Gender*						
Male	1					
Female	0.83	0.36–1.91	0.67			
*Marital status*						
Single	1					
Married	1.63	0.56–4.71	0.37			
Divorced/widow/widower	0.92	0.27–3.13	0.9			
*Education*						
Diploma and higher	1			1		
Elementary/middle school	0.98	0.39–2.46	0.97	0.99	0.39–2.54	0.98
Illiterate	3.0	0.82–10.98	0.1	3.46	0.89–13.48	0.07
*Job*						
Unemployed	1					
Employed	1.24	0.54–2.84	0.6			
*Income*						
< US $ 60	1					
> US $ 60	1.3	0.57–2.98	0.54			
*CD4 count*						
350 and less	1					
Higher than 350	0.76	0.38–2.02	0.76			
*Viral load*						
Less than 50	1					
50 and higher	0.7	0.29–1.69	0.43			
*Risk factor*						
No	1					
Yes	0.92	0.4–2.12	0.84			
*Disease history*						
Less than 5 years	1					
5–10	0.86	0.29–2.54	0.78			
10–15	1.04	0.29–3.66	0.96			
15 and higher	1.24	0.41–3.78	0.7			
*Having symptoms in the past 4* *weeks*						
No	1			1		
Yes	3.36	1.18–9.58	0.02	3.63	1.24–10.62	0.02

1: reference group

### Discussion

Our study showed that 75.4% of participants had an appropriate level of ART adherence. The main reasons for ART non-adherence included forgetfulness in taking ART (26.7%), high number of pills, and transportation problems (13.3% each).

Similar to our study, the ART adherence rates in HIV patients in Africa and Brazil were reported in the range of 66-77% [7, 13]. Contrary to the present study, the ART adherence of PWHIV in Kenya and India was reported at a low level [5, 14]. The culture of each society and the rate of AIDS-related stigma can affect the attitude of patients towards the ART adherence [9].

Similar to our study, Fonsah et al. [13] showed that the main reason for ART non-adherence was the patients’ forgetfulness. However, Bam et al. mentioned the side effects of ART as another major cause of ART non-adherence in these patients [6]. Differences in the study tools may lead to these discrepancies between results of the present study and those reported by Bam et al. [6]. Another factor to justify the variety among the results can be attributed to the fact that some questions of the instrument used by Rasoulinezhad et al. were different from those of the questionnaire used in our study [4].

Results of the present study showed that the odds ratio of poor adherence among symptomatic people was 3.63 higher than that of the asymptomatic individuals in the past 4 weeks. Nyamathi et al. believed that PWHIV would adhere to their drug regimen in the case that they saw improvements in their recovery process [5]. The current study showed no relationship among CD4+ T-cell count, viral load, and ART adherence. However, Fonsah [13] and Focà et al. [15] showed that the odds of ART non-adherence increased in PWHIV by decrease of CD4+ T-cell levels and increase of the viral load.

According to Fonsah et al. [16], individuals’ ART regimen changes by increase of the viral load and this leads to ART non-adherence. However, this issue was confirmed in no study.

Probably, the time and place of the research and the study population are effective factors in causing variety among the results.

In the present research, no significant relationship was observed between the ART adherence and gender. Contrary to the present study, Fonsah et al. showed that the ART adherence was lower in men than women [13]. African women similar Iranian women often get the HIV-test during pregnancy. One of the reasons for this heterogeneity may be the difference in social status and the role of women and men in different societies.

Furthermore, no relationship was observed between the ART adherence and education in the present study, which is consistent with the results of studies by Mukui et al., Nyamathi et al. and Fonsah et al. [5, 13, 14]. However, Bam et al. [6] showed that the ART adherence increased with higher levels of education. A reason for this contradiction is the difference in the study population and the individuals’ level of health literacy in each society. In the present study, no relationship was seen between marital status and ART adherence, which is consistent with the study by Mukui et al. [14].

### Conclusions

Educational interventions are recommended to reduce patients’ forgetfulness. The number of pills should also be decreased by combining pills together. Moreover, PWHIV are required to be supported financially and mentally by the governments and communities. The medications should be standardized in color and designs, so that similar forms be produced and used throughout the world.

## Limitations

- The outcome variable was totally determined based on the study participants’ memory and knowledge.
- The low number of samples reduced the statistical power of the study.

## Abbreviations

PWHIV: people living with HIV
cART: combined antiretroviral therapy
ACTG: The AIDS Clinical Trial Group
